# Ideal L2 Self, Self-Efficacy, and Pragmatic Production: The Mediating Role of Willingness to Communicate in Learning English as a Foreign Language

**DOI:** 10.3390/bs13070597

**Published:** 2023-07-16

**Authors:** He Yang, Zheyu Lian

**Affiliations:** College of Foreign Languages and Cultures, Xiamen University, Xiamen 361005, China

**Keywords:** ideal L2 self, self-efficacy, WTC, pragmatic production, individual difference

## Abstract

The role that individual difference factors play in pragmatic learning behavior has received increasing attention in second-language (L2) pragmatics. However, there is a dearth of studies exploring the relationship between learners’ motivational variables and their pragmatic production. To address this gap, the present study aims to examine a model of the ideal L2 self, self-efficacy, willingness to communicate (WTC), and pragmatic production among English-as-a-foreign-language (EFL) learners. The study also seeks to explore the mediating role of WTC within this structural model. For this purpose, a total of 427 undergraduate students at a public university in China were recruited for an online survey. The structural validity of the questionnaires was established using a confirmatory-factor analysis, while the hypothesized structural relations between the variables were tested through structural-equation modeling. The results demonstrated that self-efficacy and WTC significantly and directly predicated pragmatic production. Nevertheless, the ideal L2 self influenced pragmatic production indirectly, through the mediation of WTC. The study concludes by providing implications for teaching and by offering suggestions for future research.

## 1. Introduction

Pragmatic competence is generally recognized as a critical aspect of second-language (L2) teaching and learning [1,2]. However, developing pragmatic competence can be challenging for L2 learners of all levels. A lack of pragmatic knowledge in daily communication sometimes causes even advanced users of L2 to use a language inappropriately [3,4]. It is generally believed that pragmatic competence entails both productive and receptive pragmatic competences [5,6], as during the process of interaction, language users are both speakers and listeners. Pragmatic competence is a multi-dimensional and multi-layered construct [7], and most L2-pragmatics studies tend to investigate only one aspect of learners’ pragmatic competence—either pragmatic production or perception.

In recent decades, L2-pragmatics research shifted its focus from learners’ pragmatic performance to pragmatic development, and then to the factors influencing pragmatic learning. Production studies often examine learner production across diverse influential factors [8]. These factors include both external learning environments and learners’ inner characteristics. Previous studies examined the influence of learning environments (study abroad vs. study at home) on pragmatic production (e.g., [9,10], whereas more recent studies have increasingly focused on the roles learners’ individual differences (ID) play in pragmatic learning [11,12,13,14,15]. Furthermore, as pragmatics is conceptually complex, recent L2-pragmatics research has tended to examine multiple ID variables simultaneously [11,16]. These studies provide valuable contributions to our understanding of pragmatic learning, addressing the need to investigate the impact of ID variables on pragmatic competence [17]. Efforts to investigate the effects of ID factors on pragmatic production have somewhat narrowed the gap between L2-pragmatics research and mainstream SLA research.

Language-learning motivation, a key ID variable, influences the processes and outcomes of L2 learning [18]. However, to date, little published research has investigated the potential link between learners’ L2 motivation and their pragmatic production (for exceptions, see [14,19,20]). Further, motivational variables, such as the ideal L2 self, self-efficacy beliefs, and willingness to communicate (WTC) have rarely been addressed in a single study. The simultaneous investigation of these motivational ID variables may provide illuminating insights into the factors influencing learners’ pragmatic learning and behaviors. Therefore, this study aims to investigate the extent to which Chinese-university EFL learners’ ideal L2 selves and self-efficacy beliefs influenced pragmatic production and whether WTC mediated these connections. Moreover, this study is novel in combining these variables, since no previous studies on pragmatic production have explored the possible correlations between them.

## 2. Literature Review

### 2.1. Pragmatic Production and Willingness to Communicate

Pragmatic production generally denotes the ability of a language learner to perform appropriate speech acts in various situational contexts [21]. Pragmatic-production studies often examine how learners generate speech acts, taking into consideration various ID variables, such as aptitude [22], motivation [14,19,20], personality [23], proficiency [24,25], and gender [26] (see [27], for an overview).

Willingness to communicate (WTC), as an ID variable in SLA, has received considerable attention for decades. The notion of willingness to communicate (WTC) in L2 is generally defined as “a readiness to enter into discourse at a particular time with a specific person or persons, using an L2” [28] (p. 547). It is suggested that WTC in L2 is situated in nature [29,30], and that it may change when learners interact with their environments. Therefore, contextual influences have been extensively examined in WTC research [31]. In light of the importance of context in pragmatics, it is generally believed that WTC research and pragmatics research may be linked [32].

To the best of our knowledge, however, the potential links between WTC and pragmatic competence have seldom been examined. A notable example among the few existing studies is Hosseinpur and Nevisi’s investigation of the relationship between learners’ L2 WTC, learner subjectivity, anxiety, and pragmatic knowledge [19]. Their research confirmed the correlation between WTC and pragmatic production in Iranian EFL learners. There was a direct and strong correlation between the two variables among all the 140 participants, indicating that WTC played a critical role in fostering the learners’ pragmatic competence. In the same vein, [16] analyzed how WTC was correlated with receptive pragmatic competence among learners of Chinese as a second language. However, in their study, no correlation was found either between WTC and pragmatic awareness or between WTC and pragmatic comprehension. In light of previous research, we can tentatively hypothesize that WTC has different effects on productive and receptive pragmatic competences. Therefore, it is justifiable that WTC should be closely explored, as both a predictor and a mediator of pragmatic competence.

### 2.2. Ideal L2 Self

The ideal L2 self is the central concept in the L2 Motivational Self System (L2MSS) framework developed by Dörnyei [29,33]. This construct reflects how individuals envision their ideal future selves as L2 users. By developing a clearer image of their ideal L2 selves, L2 learners become more motivated to reduce the gap between their current and future selves [33]. Thus, it would appear that a vivid ideal L2 self can motivate the learning of a target language. Some studies found positive associations between ideal the L2 self and learners’ pragmatic production [20] and L2 WTC [34,35]. However, no studies have investigated the relationships between these three variables simultaneously in a single analysis.

An emerging area of research in L2 pragmatics is the impact of ID variables on pragmatic learning [8,17]. Extensive research has been conducted on motivation, and its influence on pragmatic competence has been empirically established [12,13,15,20,36]. A recent study on the links between all the components of L2MSS and productive pragmatic competence discovered a positive association between learners’ levels of pragmatic production and the ideal L2 self [20]. The findings showed that learners with a clearer picture of the correct way to communicate in their target language may be more sensitive to pragmatic and sociocultural aspects, and may be more likely to benefit from pragmatic learning opportunities. As a result, learners who possess a higher level of ideal L2 self might develop better pragmatic production. This discovery corroborated Dörnyei’s suggestion’s that higher self-guided scores are linked to greater success in L2 achievement [29,33].

Empirical evidence suggests that the ideal L2 self is one of the antecedents of L2 WTC in a broad range of EFL contexts. As far back as 2013, Munezane [37] found that a new predictor (i.e., the ideal L2 self) had a significant impact on WTC in a Japanese EFL context. According to her argument, studying ideal L2 self is “worthwhile” in EFL contexts, where native English speakers are less likely to interact with students (p. 177). Lee and Lee [34] observed that Korean EFL university students with stronger ideal L2 selves exhibited levels of L2 WTC in both focus-group discussions and individual interviews. More recent studies also confirmed the direct and positive correlation between the two constructs in Chinese and Iranian EFL contexts, in which emotional variables, such as boredom [35], shyness and grit [38] were examined as mediators.

### 2.3. Self-Efficacy Beliefs

Self-efficacy refers to a person’s judgement or perception regarding their ability to fulfill a specific task based on their skills [39]. It is believed that self-efficacy affects the motivation of L2 students to work hard and persevere in reaching their learning goals [40,41,42]. Further, the level of self-efficacy of an individual is considered to influence their learning outcomes [43,44].

Studies have revealed a positive association between learners’ self-efficacy and academic achievement in the acquisition of foreign languages [45,46,47,48]. For example, Bai, Chao, and Wang [45] highlighted the role of students’ self-efficacy and social support in accounting for their English-learning achievements. Their results showed a positive correlation between self-efficacy and L2 achievement. Moreover, self-efficacy and parental support were significant predictors of English-learning achievement among Hong Kong secondary students. Furthermore, Kim et al. [47] found that students with stronger self-efficacy profiles employed their self-regulated learning strategies more frequently and achieved greater success in learning English than their peers with lower self-efficacy profiles. A mixed-methods study, making it particularly pertinent to the present study, revealed that Chinese university students’ self-efficacy beliefs regarding language proficiency hindered their pragmatic learning, whereas students’ high self-efficacy beliefs regarding L2 cultural knowledge were reported to greatly assist them in recognizing L2 speakers’ intentions (i.e., one aspect of pragmatic comprehension) during communication [36].

The relationship between self-efficacy and the ideal L2 self was investigated in a few studies, and a positive correlation was discovered [49,50]. For example, Busse’s study of first-year students of German found that the students’ task-based self-efficacy was strongly correlated with their ideal L2 self [49]. She argued that by attending to the students’ self-efficacy beliefs, their ideal L2 selves could also be nurtured, given the significant correlation between these two variables. Furthermore, according to Kim [50], self-efficacy and the ideal L2 self are correlated among Korean university students majoring in English-related studies. Additionally, self-efficacy was found to be more powerful than experience abroad in the students’ predictions of their ideal L2 selves.

The present study also examines the association between self-efficacy and WTC, which has received less attention than the link between WTC and the ideal L2 self. Among the few studies to research this was Saka and Merç’s investigation [51], which explored the relationship between Turkish EFL learners’ self-efficacy, L2 WTC, and linguistic self-confidence and demonstrated that the learners’ self-efficacy and WTC were positively correlated. Together, these findings suggest that self-efficacy beliefs exert a crucial and complex effect on L2 learners’ learning behaviour, L2 achievements, and pragmatic competence.

**Figure 1:**
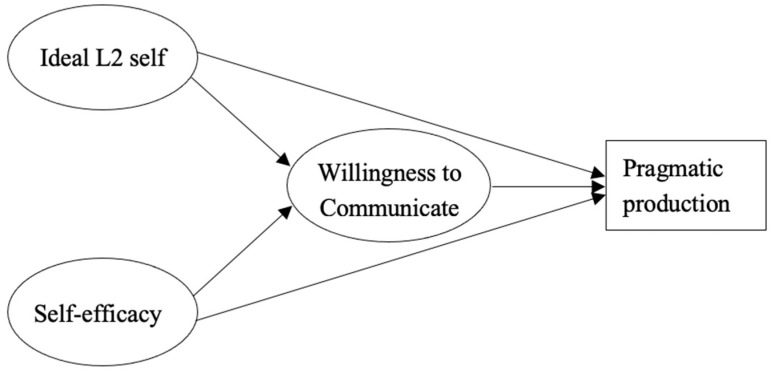
The hypothesized structural model.

As reviewed above, no previous studies have simultaneously investigated the relationship between the ideal L2 self, self-efficacy beliefs, and pragmatic production with the construct of WTC serving as a mediating variable. Consequently, a further investigation into the possible associations between the aforementioned variables appears to be essential. Therefore, a structural model of the ideal L2 self, self-efficacy beliefs, WTC, and pragmatic production was hypothesized based on the theoretical foundations of the constructs and the literature review. In Figure 1, the hypothesized model and its paths are graphically represented.

The hypotheses derived from this model include the following:

**Hypothesis 1.**  *The ideal L2 self has a positive and significant effect on pragmatic production.*

**Hypothesis 2.**  *Self-efficacy has a positive and significant effect on pragmatic production.*

**Hypothesis 3.**  *The WTC has a positive and significant effect on pragmatic production.*

**Hypothesis 4.**  *The ideal L2 self has an indirect effect on pragmatic production through the mediation of WTC.*

**Hypothesis 5.**  *Self-efficacy has an indirect effect on pragmatic production through the mediation of WTC.*

## 3. Methodology

In this study, a quantitative research design is used to examine the antecedents of EFL learners’ L2 pragmatic production. The variables examined are mainly motivational variables, such as ideal L2 self, self-efficacy, and WTC.

### 3.1. Participants

A total of 427 first- and second-year undergraduate students were recruited from a Chinese public university, of whom 172 were male and 255 female (M = 19.8, SD = 2.7). Participants were selected using a convenience-sampling procedure, and their majors included Advertising (11.7%), Chemistry (30.1%), Education (19.8%), Finance (16.2%), and Mathematics (26.7%). All participants took the required two-credit College English courses, which focused on developing the four language skills, with an emphasis on listening and speaking. The participants were classified as students with an intermediate proficiency level based on the placement test at the beginning of their college studies. All participants’ mother tongue was Mandarin Chinese, and they reported no study-abroad experience prior to participating in this study.

### 3.2. Instruments

A composite online survey was used to collect data. The survey consisted of four parts: three Likert scales tapping into the variables of ideal L2 self, self-efficacy, and WTC, and a discourse-completion task (DCT) measuring learners’ pragmatic production. Finally, demographic queries (e.g., age, gender, major, and study-abroad experience) were included. The scales are described below (refer to Supplementary Materials for the complete questionnaire items of the survey).

#### 3.2.1. Ideal L2 Self Scale

Learners’ ideal L2 self was measured using eight items originally developed by Taguchi, Magid, and Papi [52] for English learners in Japan, China, and Iran. The self-report scale measured learners’ perceptions of their ideal selves as English users. Each item was rated on a Likert scale ranging from 1 for “strongly disagree” to 6 for “strongly agree.” The item, “I imagine myself speaking English with international friends or colleagues” is an example of this scale.

#### 3.2.2. Self-Efficacy Scale

The items used to assess learners’ self-efficacy beliefs were adapted from Busse [49]. The original scale consisted of 14 items measuring learners’ self-efficacy beliefs while they were learning German. For the purpose of this study, some items were removed, such as those related to translation self-efficacy. Finally, the items were trimmed to eight, focusing on the four skills and classified into two subscales: reading-and-writing self-efficacy (4 items, α = 0.88), and listening-and-speaking self-efficacy (4 items, α = 0.89). Each item was scored on a Likert scale, with 1 being “strongly disagree” and 6 being “strongly agree.” An example item was: “How confident are you that you will be able to write an essay in English by the end of this academic year?”.

#### 3.2.3. WTC Scale

Participants’ WTC was assessed using the items developed by Joe, Hiver, and Al-Hoorie [53] for Korean L1 respondents. This scale was originally adapted from Pae’s WTC [54] and consisted of 12 items categorized into three subscales: WTC in L2 with friends (4 items, α = 0.85), acquaintances (4 items, α = 0.85), and strangers (4 items, α = 0.83). A 6-point Likert scale was used for the items, with 1 representing “strongly disagree” and 6 representing “strongly agree”. The following is an example of an item on this scale: “I would like to talk in English in a large meeting of acquaintances.”.

#### 3.2.4. Discourse-Completion Task

This study operationalized pragmatic production as the appropriate production of speech acts (e.g., complaints) in different situations. Appropriateness was measured using the discourse-completion task (DCT) designed by Yang [14]. Her DCT focused on the speech act of complaints, while social distance and social power were examined as contextual variables. The DCT consisted of six scenarios, each accompanied by an explanation of the situation, along with a gap designed to elicit responses from participants based on their imaginary interactions. A sample DCT scenario was as follows:

You order a drink in a restaurant. When the waiter brings you the drink, he spills it all over you. Your new shirt is stained. The waiter says, ‘Oh, I’m really sorry about that!’.

You say: ________________________.

Although DCT is often criticized for not being able to show the actual wording used in real-life situations [55], it allows researchers to manipulate contextual variables (e.g., social status and social distance) in designed scenarios [56]. Moreover, it helps participants demonstrate their pragmatic-knowledge and speech-act repertoires [57].

As the aim of the investigation into learners’ pragmatic production was to ascertain participants’ offline knowledge of pragmalinguistics when interacting with a variety of interlocutors, such as those of equal or higher status, in different contextual circumstances, DCT was still a very useful instrument for this study.

### 3.3. Procedure

Data were collected in the early spring of 2023.

An independent researcher familiar with both languages and the principles of questionnaire construction translated the scales measuring the three motivational variables into students’ L1 (Chinese), and the first author translated them back into English. The self-report scales, as well as the DCT assessing learners’ L2 pragmatic production, were transformed into a Sojump online survey. The author of the present study asked several College English course instructors to administer the online questionnaire during their class time. By scanning the survey’s QR code using their cell phones, students indicated that they agreed to take part in the survey voluntarily. All participants responded to the questions in the four parts of the online survey. For the Likert-scale questions, they selected from the provided choices, whereas for the DCT, they typed in their responses. Moreover, they were told that their participation would not affect their grades for the term.

### 3.4. Data Analysis

The analysis of data involved two procedures. First, data were examined using SPSS 25 to account for missing values, outliers, normality, and multicollinearity. Second, in order to investigate pragmatic production and the three motivational variables, we applied SEM using the AMOS 24. Confirmatory-factor analyses (CFA) were conducted for each scale to assess the structural validity of the measurement model. Next, SEM was used to test the hypothesized model. Our overall model fit was evaluated based on the most commonly recommended indices in SEM publications. Apart from Chi-square divided by degree of freedom (χ^2^/df), additional indices were also reported, including the comparative fit index (CFI), the goodness-of-fit index (GFI), the Tucker–Lewis index (TLI), and the root-mean-square error of approximation (RMSEA). Models were considered to fit when χ^2^/df < 5, CFI and TLI > 0.90, and RMSEA < 0.08 [58].

As in previous research, DCT answers were coded on a scale ranging from 1 for “most inappropriate” to 6 for “most appropriate” by two native-English-speaking raters. Based on their intuitions, the two raters independently scored participants’ overall performances of their DCT by assessing how they would feel about the participants’ response if they were the hearers in these complaint scenarios. Participants’ scores were calculated by averaging the ratings of two raters.

## 4. Empirical Findings

### 4.1. Preliminary Analyses

The SPSS 25 was used to screen the data before the hypothesized model was tested. In order to deal with missing data, we used the Expectation-Maximisation (EM) algorithm. The missing data were processed using multiple imputation, resulting in a final sample size of *N* = 419. A Shapiro–Wilk test was conducted to assess the normality of the data, and the results indicated that all the variables (ideal L2 self, self-efficacy beliefs, WTC, and pragmatic production) were normally distributed. No variables required transformation to achieve normality. An overview of the descriptive statistics is presented in Table 1, including the means, standard deviations, skewness, kurtosis, and Cronbach’s alpha values. THE data analysis revealed that all of the computed coefficient alphas for the scales were greater than 0.85, confirming that their internal consistency was appropriate [59], and that the skewness and kurtosis measures were within the acceptable ranges [60].

In the subsequent step, in order to check the structural validity of the measurement model, confirmatory-factor analyses (CFA) were conducted for each of the scales. The results of the three CFAs showed that the theoretical concepts for measuring the latent variables (“ideal L2 self”, using eight items, “self-efficacy”, using eight items, and “willingness to communicate”, using twelve items) were supported by the data (see Table 2). The indicators, including CMIN/DF, GFI, CFI, and RMSEA, all fell within the reference range, and all the standardized regression coefficients were above 0.7. Therefore, the three measurement models were deemed stable and reliable.

Next, all the questionnaire scales were subjected to CFA to test the measurement model. The construct reliability and the average variance extracted from the measurement model are presented in Table 3, indicating that the model demonstrated satisfactory convergent and discriminant validity.

### 4.2. SEM Analyses

To further explore the relationships between the latent variables, we applied SEM to process the data. A comparison was drawn between the three models of direct effects, full mediation, and partial mediation, and the findings showed that the partial-mediation model produced more suitable fitness measures (see Table 4). Based on the partial mediation model, the fitness indices were χ^2^(367) = 712.215, *p* < 0.001, χ^2^/df = 1.941, *p* = 0.000, CFI = 0.963, TLI = 0.959, RMSEA = 0.047, and PCLOSE = 0.787.

Furthermore, Table 5 presents the path estimates for the structural models. Surprisingly, the ideal L2 self did not have a direct effect on pragmatic production, rejecting Hypothesis 1. Nevertheless, self-efficacy had a positive effect on pragmatic production (β = 0.237, *p* < 0.05), confirming Hypothesis 2. Moreover, the WTC positively affected pragmatic production significantly (β = 0.329, *p* < 0.01), confirming Hypothesis 3.

**Figure 2:**
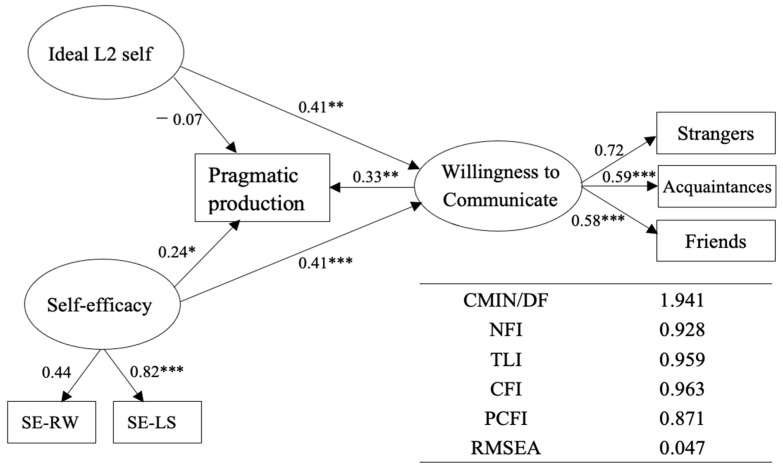
Path results of the structural model. Note: * *p* < 0.05, ** *p* < 0.01, *** *p* < 0.001.

Baron and Kenny’s method [61] was then used to examine the WTC’s mediating role. The proposed model met all of the requirements. The partial-mediation model (see Table 5) indicated that the ideal L2 self had no significant impact on pragmatic production; however, self-efficacy did. Additionally, the ideal L2 self indirectly influenced pragmatic production through the WTC 0.135 (0.41* × 0.33), confirming Hypothesis 4. Similarly, self-efficacy significantly affected pragmatic production indirectly through the WTC 0.135 (0.41 × 0.33), confirming Hypothesis 5. An overview of the path estimates is shown in Figure 2 (refer to Supplementary Materials for the full mode).

## 5. Discussion

Several significant findings were observed based on the hypothesized model and the proposed hypotheses. First, the ideal L2 self did not have a direct impact on pragmatic production, rejecting Hypothesis 1. This finding indicated that learners with the internalized and self-motivated desire to achieve proficiency might not develop better pragmatic production. This finding is inconsistent with that of Yang and Wu [20], who reported that the ideal L2 self was positively correlated with pragmatic production. The differences between these results could be explained by the fact that different statistical analyses were employed. In Yang and Wu’s study [20], bivariate correlations were conducted to measure the association between the ideal L2 self and pragmatic production, while in the present study, a structural-equation model was tested to examine the causal relationship between the two constructs. Correlation measures the strength and direction of a relationship between two variables, but it does not provide information on causation or the ability to predict one variable based on another, whereas prediction requires additional analyses, such as a regression analysis, to determine the extent to which one variable can be used to predict another [62].

Second, the findings indicated that the participants’ self-efficacy predicted pragmatic production positively and significantly, supporting Hypothesis 2. Moreover, self-efficacy was also found to influence pragmatic production through the mediation of the WTC, thus partially supporting Hypothesis 5. The analysis revealed that learners’ self-efficacy beliefs directly affected their L2 pragmatic production regardless of the levels of their WTC in L2, suggesting that L2 self-efficacy was an important predictor of L2 productive pragmatic competence among Chinese university EFL learners. The findings of this research indicate that English self-efficacy has a significant impact on L2 results, particularly pragmatic performance, which is consistent with previous studies [36,45,46,48]. Furthermore, the findings lend support to Yang’s proposition [36] that learners’ L2-self-efficacy beliefs might affect their pragmatic learning behaviour and pragmatic achievements. The beliefs that learners hold about their ability to proficiently use their target language might enhance their willingness and confidence to engage in more language practice, which could, in turn, lead to an increase in their pragmatic competence.

Third, the SEM results confirmed Hypothesis 3 by revealing that WTC positively affected learners’ pragmatic production. It appears that learners with higher L2 WTC are more inclined to take risks, behave proactively, and engage in communication [30]. Such learners may recognize that both grammatical knowledge and pragmatic knowledge are essential components of effective communication; therefore, they might become more motivated to equip themselves with stronger pragmatic skills. The results obtained in this study support the findings in the study by Hosseinpur and Nevisi [19], which reported a positive correlation between WTC and request production among Iranian learners of English. In contrast, Lv, Ren, and Li [16] found no correlation between WTC and receptive pragmatic competence (including pragmatic awareness and comprehension) among L2 learners of Chinese. Therefore, we can tentatively conclude that L2 WTC has different effects on productive and receptive pragmatic competences. In this regard, the current study supports Taguchi and Roever’s claim [17] that receptive and productive pragmatic competences fall into different domains and are influenced differently by L2 motivation.

Finally, the results showed that the ideal L2 self affected pragmatic production through the mediation of the WTC, supporting Hypothesis 4. This finding partially supports Hypothesis 1. The results suggest that university students with a more vivid image of themselves as competent L2 users are more inclined to communicate in their target languages. Our findings indicate that WTC did play a mediating role in the relationship between the ideal L2 self and pragmatic production. This result is in line with the empirical findings in previous studies (e.g., [34,35,37,38,63], where language learners with more vivid images of themselves as competent L2 users had a greater willingness to communicate in their target languages. As discussed above, students with higher levels of WTC are likely to recognize that communication requires both grammatical and pragmatic knowledge. Consequently, they tend to endeavour to improve their pragmatic skills in order to initiate and maintain effective communication.

## 6. Conclusions

The purpose of this study was to explore whether the ideal L2 self, self-efficacy, and WTC had special roles to play in influencing L2 pragmatic production. In the study, we found that self-efficacy significantly influenced pragmatic production, either directly or indirectly, through the mediation of the WTC, while the ideal L2 self did not have a similar effect. The ideal L2 self influenced pragmatic production only through the mediating role of the WTC. Thus, the learners with more vivid images of themselves as competent L2 users did not exhibit better productive pragmatic competence, but the mediating effects of the WTC facilitated the influence of this construct on pragmatic production. Overall, the SEM analysis confirmed that the WTC has a significant mediating role in influencing learners’ pragmatic production. Another important finding of this study was that both the ideal L2 self and self-efficacy beliefs were the antecedents of the WTC, and that the WTC was a significant predictor of pragmatic production.

Based on the current study, it appears that self-efficacy plays a significant role in improving pragmatic performance. This means that language teachers should plan and prepare lessons that help students to develop a sense of improved self-efficacy by successfully completing language tasks. For instance, teachers can regularly provide various opportunities for students to enhance their personal L2 self-efficacy, tailored to their progress in L2 pragmatic learning. When students feel more confident about their learning, they are more likely to engage with materials and achieve their learning goals. Teachers can facilitate this process by drawing students’ attention to sociocultural aspects, explaining how linguistic forms relate to their functions and context, guiding their understanding of the cultural meanings behind pragmatic phenomena, offering continuous feedback, and supporting learners in making their own pragmatic choices based on their newly acquired pragmatic competence and awareness [64]. Additionally, it is worthwhile to consider the creation of a syllabus that gives students more opportunities to develop their pragmatic abilities in English classes.

A few limitations in this study should be noted. Firstly, the sample data collected were exclusively from students at a prestigious public university, so they are not representative of EFL students at other Chinese universities. In future studies, a more diverse sample of data may be necessary to identify the effects of the examined constructs on L2 pragmatic production. Furthermore, this study relied solely on quantitative data; however, qualitative data would be helpful in shedding light on the complex roles that various influential variables play in pragmatic learning. A combination of qualitative and quantitative methods is recommended for in-depth research to take advantage of both approaches’ strengths [65]. Despite these limitations, the present findings support the notion that motivational variables play a vital role in L2 pragmatic learning.

## Figures and Tables

**Table 1:** Descriptive statistics (*N* = 419).

	Mean	SD	Skewness	Kurtosis	Cronbach’s α
Ideal L2 self	3.750	1.335	−0.372	−0.942	0.96
Self-efficacy	3.689	1.010	−0.328	−0.244	0.87
Willingness to communicate	3.372	0.916	0.184	0.585	0.89
Pragmatic production	3.563	1.598	−0.062	−1.041	-

**Table 2:** Measurement model of the motivational variables.

	χ^2^	Df	χ^2^/df	GFI	CFI	RMSEA
Ideal L2 self	50.46	20	2.52	0.97	0.99	0.060
Self-efficacy	63.51	19	3.34	0.96	0.98	0.075
Willingness to communicate	150.14	51	2.94	0.94	0.97	0.068

**Table 3:** Convergent and discriminant validity of the constructs.

Construct	Convergent Validity		Discriminant Validity			
	CR	AVE	1	2	3	4
1. Ideal L2 self	0.96	0.77	0.88			
2. Self-efficacy	0.96	0.75	0.26 **	0.86		
3. Willingness to communicate	0.97	0.74	0.40 **	0.31 **	0.86	
4. Pragmatic production	-	-	0.21 **	0.35 **	0.34 **	1.00

Note: CR = composite reliability, AVE = average variance extracted. Values in the diagonal are the square roots of their respective AVEs. ** *p* < 0.01.

**Table 4:** Fitness indices of the models.

Model	χ^2^/df	Δχ^2^	CFI	TLI	RMSEA
Direct-effects model	2.191	-	0.976	0.972	0.053
Full-effects model	1.954	469.051	0.963	0.959	0.048
Partial-effects model	1.941	8.773	0.963	0.959	0.047

Note: Δχ^2^ shows differences between the model and the subsequent model. CFI = comparative fit index, TLI = Tucker–Lewis index, and RMSEA = root-mean-square error of approximation.

**Table 5:** Path estimates of the models.

Path	Standardized Path Coefficients (CR)		
	Direct Model	Full-Mediation Model	Partial-Mediation Model
Ideal L2 self → pragmatic production	0.053 (0.843)		−0.066 (−1.021)
Self-efficacy → pragmatic production	0.4 (4.528 ***)		0.237 (2.544 *)
Ideal L2 self → WTC		0.407 (5.101 ***)	0.406 (5.084 ***)
Self-efficacy → WTC		0.414 (4.342 ***)	0.406 (3.976 ***)
WTC → pragmatic production		0.445 (6.634 ***)	0.329 (3.126 **)

Note: * *p* < 0.05, ** *p* < 0.01, *** *p* < 0.001.

## Data Availability

Not applicable.

## Supplementary Materials

The following supporting information can be downloaded at: https://www.mdpi.com/article/10.3390/bs13070597/s1. Figure S1: full model; questionnaire items.
