# Supplementary material for: Ideal L2 Self, Self-Efficacy, and Pragmatic Production: The Mediating Role of Willingness to Communicate in Learning English as a Foreign Language
Source: Behav Sci (Basel). 2023 Jul 16;13(7):597. doi: 10.3390/bs13070597 (PMC10375955; doi:10.3390/bs13070597)
Supplement: Supplementary file 1 [file behavsci-13-00597-s001.zip › behavsci-2446883-supplementary.pdf]

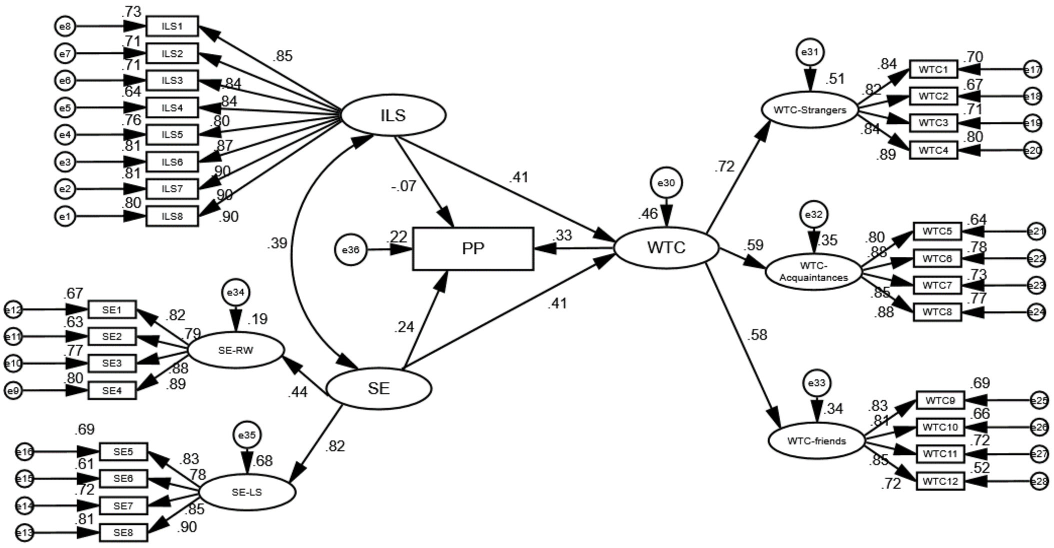

Figure S1. Full model

Questionnaire items

Ideal L2 self

I can imagine myself living abroad and using English effectively for communicating with the locals.
I imagine myself speaking English as if I were a native speaker of English.
I imagine myself speaking English with international friends or colleagues.
Whenever I think of my future career, I imagine myself using English.
I imagine myself studying in a university where all my courses are taught in English.
I imagine myself writing English e-mails fluently.
I imagine myself being a person known as a fluent speaker of English.
I imagine myself living and making friends in a modern community, using English.

我可以想象自己生活在国外，有效地使用英语与当地人交流。
我可以想象自己说英语，就像我是一个以英语为母语的人。
我可以想象自己与国际朋友或同事说英语。
每当我想到我未来的职业，我就想象自己会使用英语。
我可以想象自己在一所大学里学习，所有课程都是用英语授课。
我可以想象自己能流利地写英文电子邮件。
我可以想象自己成为一个以讲流利英语著称的人。
我可以想象自己使用英语在一个现代社区生活和交朋友。

Self-Efficacy

How confident are you that by the end of this academic year you will be able to

Reading and writing self-efficacy

- write an essay in English?

- write a short summary of a newspaper article in English?
- write your opinion about an article in English?
- read and understand a newspaper article?

Listening and speaking self-efficacy

- listen to and understand a lecture in English?
- listen to and understand an English film?
- speak about a film with a native speaker?
- discuss a newspaper article with a native speaker?

我有多大信心在本学年结束时，我能够

- 用英语写一篇作文？
- 用英语写一篇报纸文章的简短摘要？
- 用英语写出我对一篇文章的看法？
- 阅读并理解一篇报纸文章？
- 聆听并理解英语讲座？
- 观看并理解一部英语电影？
- 与母语人士谈论一部电影？
- 与母语人士讨论一篇报纸文章？

Willingness to communicate

Willingness to communicate with strangers

I would like to present a talk in English to a group of strangers.
I would like to talk in English in a small group of strangers.
I would like to talk in English with a stranger while standing in line.
I would like to talk in English in a large meeting of strangers.
我想用英语向一群陌生人发表演讲。
我想在一小群陌生人中用英语交谈。
我想在排队时与陌生人用英语交谈。
我想在陌生人的大型会议上用英语交谈。

Willingness to communicate with acquaintances

I would like to talk in English with an acquaintance while standing in line.
I would like to talk in English in a large meeting of acquaintances.
I would like to talk in English in a small group of acquaintances.
I would like to present a talk in English to a group of acquaintances.
我想在排队时与熟人用英语交谈。
我想在一个大型的熟人聚会中用英语交谈。
我想在一小群熟人中用英语交谈。
我想用英语向一群熟人发表演讲。

Willingness to communicate with friends

I would like to talk in English in a large meeting of friends.
I would like to talk in English with a friend while standing in line.

I would like to present a talk in English to a group of friends.
I would like to talk in English to a small group of friends.
我想在朋友的大型会议上用英语交谈。
我想在排队时与朋友用英语交谈。
我想用英语向一群朋友发表演讲。
我想用英语与一小群朋友交谈。

Discourse Completion Task

Directions: Please read the scenarios before you answer them. Imagine that you are in the situation as described by each scenario and respond spontaneously as you do in face-to-face interaction.

Example: You are having coffee with a friend before your seminar. You want to check whether you have to leave soon, but you realize that you don’t have your watch with you. You ask your friend for the time.

You say:

You are handed back a paper by your professor. However, you are startled by your grade and feel that you have been marked down for disagreeing with the professor’s point of view rather than on any flaws in your content and analysis. You are particularly upset since you have spent weeks researching this paper and feel the professor has ignored your effort through simple bias. You decide you must speak to him/her about this. So, after class, you go to the professor during office hours.

You say:

You are a student of a university. You asked for your tuition fee to your father three days ago. Your father promises that you will receive the money today. Now you are going to your campus to pay for your tuition fee as today is the deadline for payment. Unfortunately, when you ask your father for the money, he says that he has forgotten to withdraw the money from the bank but he will give you the money this afternoon or tomorrow morning. You complain to your father about this.

You say:

You are living in a dormitory. It is 11:30 p.m. and you are still studying for the final exam that will take place tomorrow morning. You hear the neighbour next door playing rock music. The music is getting louder and louder, disrupting your concentration. You go to your neighbour next door to complain about it.

You say:

A friend who takes the same course as you at the university declines to share some important material for the next test, which s/he has managed to get hold of. In the past, you helped him/her many times.
You see him/her on campus and say:

You ordered a drink in a restaurant. When the waiter brings you the drink, he spills it all over you. Your new shirt is stained. The waiter said ‘Oh, I’m really sorry about that!’
You say:

You are talking on the phone to your classmate. Your 10-year-old younger brother, Peter, is playing around the house, making a lot of noise. You can hardly hear your classmate. **You:** Peter! Peter!
Peter: Yeah?
You:
